# The reaction zone is a unique plant defense found in trees: differentially expressed genes and cell wall changes

**DOI:** 10.1186/1753-6561-5-S7-P84

**Published:** 2011-09-13

**Authors:** Carl Gunnar Fossdal, Nina Nagy, Hietala Ari, Igor Yakovlev, Halvor Solheim

**Affiliations:** 1 Norwegian Forest and Landscape Institute, Norway

## 

*Heterobasidion annosum *senso lato is the most devastating pathogen of conifers such as Norway spruce in Europe. This pathogen enter Norway spruce trees trough the roots or wounds and colonizes the tree from within, growing as a saprophyte when established within the dead heartwood and acting as a necrotroph when in contact with living host tissue. We have examined the host response in Norway spruce at the molecular level as well as the responses of the pathogen.

We have studied the defense reactions toward pathogenic fungi in the ecological and economic important conifer Norway spruce from both a molecular and anatomical perspective. We have studied the host responses of the tree as well as the attack modes and genes indused by its pathogens. The disease caused by this *H. annosum* s.l. is complex as it is can act both a necrotroph and saprotroph as well as a broad host range. Twenty percent of the trees in Norwegian spruce stands tend to be infected by this pathogen and *H. annosum* s.l. typically colonize and decay the economically important wood inside the trunk. However, Norway spruce have defences against this and other pathogens and the attack can be fought off by the living bark and sapwood but not the hearthwood being composed of dead zylem.

The bark has effective defense reactions that can be induced and we have seen indications of systemic and primed defense responses but also the sapwood has defensive capabilities. The tree has a unique defense against this internal attack by forming a reaction zone; in this case the host defense is directed inwardly by the still living sapwood toward the central colonized wood. We have studied the host defense at the transcriptional level, changes in phenols and lignification and found that the speed of the host response appear to be crucial in fending off the pathogen.

